# Biochemical Comparison of dsRNA Degrading Nucleases in Four Different Insects

**DOI:** 10.3389/fphys.2018.00624

**Published:** 2018-05-28

**Authors:** Yingchuan Peng, Kangxu Wang, Wenxi Fu, Chengwang Sheng, Zhaojun Han

**Affiliations:** The Agricultural Ministry Key Laboratory of Monitoring and Management of Plant Diseases and Insects, Department of Entomology, College of Plant Protection, Nanjing Agricultural University, Nanjing, China

**Keywords:** RNA interference, nuclease, dsRNA degrading enzyme, dsRNase, fluorescence

## Abstract

Double stranded RNAs (dsRNA) degrading nuclease is responsible for the rapid degradation of dsRNA molecules, and thus accounts for variations in RNA interference (RNAi) efficacy among insect species. Here, the biochemical properties and tissue-specific activities of dsRNA degrading nucleases in four insects (*Spodoptera litura, Locusta migratoria, Periplaneta americana*, and *Zophobas atratus*) from different orders were characterized using a modified assay method. The results revealed that all insect dsRNA degrading nucleases tested showed high activity in alkaline environments at optimal Mg^2+^ concentrations and elevated temperatures. We also found that enzymes from different insects varied in terms of their optimal reaction conditions and kinetic parameters. Whole body enzyme activity differed dramatically between insect species, although enzymes with higher substrate affinities (lower *K*_m_) were usually balanced by a smaller *V*_max_ to maintain a proper level of degradative capacity. Furthermore, enzyme activities varied significantly between the four tested tissues (whole body, gut, hemolymph, and carcass) of the insect species. All the insects tested showed several hundred-fold higher dsRNA degrading activity in their gut than in other tissues. Reaction environment analysis demonstrated that physiological conditions in the prepared gut fluid and serum of different insects were not necessarily optimal for dsRNA degrading nuclease activity. Our data describe the biochemical characteristics and tissue distributions of dsRNA degrading activities in various insects, not only explaining why oral delivery of dsRNA often produces lower RNAi effects than injection of dsRNA, but also suggesting that dsRNA-degrading activities are regulated by physiological conditions. These results allow for a better understanding of the properties of dsRNA degrading nucleases, and will aid in the development of successful RNAi strategies in insects.

## Introduction

Since the first demonstration of long, double stranded RNAs (dsRNA) mediating RNA interference (RNAi) in *Caenorhabditis elegans* (Fire et al., 1998), RNAi technology has been widely used in scientific research, clinical applications, and agricultural pest control in the past decades (Baum et al., 2007; Price and Gatehouse, 2008; Bellés, 2010; Burand and Hunter, 2013; Zhang et al., 2017). RNA interference is achieved by administration of dsRNA to the target site, where it is then processed into small interfering RNA (siRNA) by Dicer enzyme and coupled with Argonaute protein to form an RNA-induced silencing complex (RISC), which combines with complementary mRNA to induce target RNA degradation (Meister and Tuschl, 2004).

The elements involved in RNAi are indispensable for successful gene silencing (Mello and Conte, 2004; Tijsterman et al., 2004). The core RNAi machineries contributing to robust RNAi responses are widely distributed among various insect species (Tomoyasu et al., 2008; Wynant et al., 2012; Liu et al., 2013; Swevers et al., 2013; Xu et al., 2013; Ghosh et al., 2014). Currently, it is difficult to successfully silence genes with dsRNA in some insects, such as lepidopterans, which are refractory to RNAi (Terenius et al., 2011). Systemic RNAi efficiency variation among different insects has been a barrier to the application of RNAi as an effective tool for functional genomic research, and also to the biological control of pests by dsRNA-expressing transgenic plants and gene-specific dsRNA insecticides (Terenius et al., 2011; Zhang et al., 2013; Kola et al., 2015; Joga et al., 2016). Systemic RNAi requires the spread of dsRNA throughout the whole body, which relies on stable transition and uptake of dsRNA (Huvenne and Smagghe, 2010). Critically, dsRNA molecules must avoid degradation by nucleases if they are to maintain long-term stability before reaching target sites and inducing a sustainable RNAi response. It is necessary to better understand the factors underlying the diversity of dsRNA degrading activities in the hemolymph and gut lumen of different insect species.

Our previous studies found that different degrees of dsRNA degradation in the gut and hemolymph were consistent with variations in RNAi efficacy among insects (Wang et al., 2016). Previous reports have demonstrated that degradation of dsRNA was a major factor limiting RNAi efficacy in lepidopteran insects (Shukla et al., 2016). In the Colorado potato beetle, *Leptinotarsa decemlineata*, which is sensitive to RNAi response by oral delivery of dsRNA, depletion of nuclease activity by interfering dsRNase (a member of the DNA/RNA non-specific endonuclease family) in the gut increased dsRNA stability and resulted in an improved RNAi response (Spit et al., 2017). In the migratory locust *Locusta migratoria*, a dsRNase was found to be responsible for the poor RNAi response associated with oral delivery of dsRNA. Knockdown of the gene encoding this gut-specific dsRNase enhanced the RNAi response (Song et al., 2017). Additional evidence has shown that the gut fluid, hemolymph, and saliva of different insects digests dsRNA, which may negatively impact RNAi efficacy (Furusawa et al., 1993; Allen and Walker, 2012; Liu et al., 2012; Garbutt et al., 2013; Christiaens and Smagghe, 2014; Ren et al., 2014; Wynant et al., 2014; Lomate and Bonning, 2016; Almeida Garcia et al., 2017; Luo et al., 2017; Singh et al., 2017).

It has been shown that dsRNA may be absorbed and spread throughout the whole insect body (Huvenne and Smagghe, 2010; Ivashuta et al., 2015). Multiple nucleases may therefore be involved in dsRNA degradation. Besides siRNA-degrading enzymes like Eri-1, which function as secondary degrading enzymes of dsRNA, two kinds of dsRNA degrading enzymes have been identified in insects, namely Dicers and dsRNases. Dicers and dsRNases are always encoded by several genes in an insect, and different insect species usually differ in terms of their expression profiles (Tomoyasu et al., 2008; Liu et al., 2012; Wynant et al., 2014; Luo et al., 2017; Song et al., 2017; Spit et al., 2017). It is difficult to compare dsRNA degrading nucleases from different species because their identities remain largely unknown. The most well-studied dsRNases are members of a large family of DNA/RNA non-specific endonucleases, which were usually found constitutively over-expressed in the insect gut, and are thought to target and degrade foreign dsRNAs (Liu et al., 2012; Wynant et al., 2014; Luo et al., 2017; Song et al., 2017; Spit et al., 2017). As mentioned above, the strong cleavage activity of widely distributed gut-specific dsRNases against dsRNA molecules has been confirmed in different insects. These enzymes are a type of secretory nuclease expressed in a variety of tissues (Liu et al., 2012; Garbutt et al., 2013; Almeida Garcia et al., 2017; Luo et al., 2017). Some are over-expressed in the gut (Garbutt et al., 2013; Wynant et al., 2014; Almeida Garcia et al., 2017; Luo et al., 2017; Song et al., 2017; Spit et al., 2017). They play key roles in lowering RNAi efficiency by degrading dsRNA into non-functional fragments. Dicer is another well-known dsRNA degrading enzyme which functions intracellularly as part of the core RNAi machinery, cutting dsRNA into siRNA within various cells to regulate gene expression during insect development (Sinha et al., 2015). These enzymes also degrade endogenous or invading dsRNAs (Marques et al., 2013). Though positive for RNAi, Dicers also degrade dsRNA into siRNA directly. The siRNA may then be degraded by other nucleases. Thus, siRNA-degrading nucleases could promote dsRNA degradation by functioning as secondary degrading enzymes (Kennedy et al., 2004; Kupsco et al., 2006; Tomoyasu et al., 2008; Han et al., 2011; Swevers et al., 2013; Xu et al., 2013; Christiaens et al., 2014; Sinha et al., 2015). Recently, intracellular siRNA-degrading Eri-1-like nucleases were found to inhibit RNAi. Interference of their expression in *C. elegans* enhanced RNAi efficacy (Kennedy et al., 2004). However, their negative function against RNAi has not been confirmed in insects. Further studies might reveal additional nucleases degrading dsRNA in various insects. Therefore, when investigating factors which affect dsRNA persistence in insects, all enzymes present in a species, known and unknown, should be taken into consideration. For this reason, in the present study we considered all the enzymes involved by using homogenate supernatants recovered, from either whole insect bodies or from selected tissues, as enzyme sources for comparison, and hope the weighted average obtained with integrated enzymes could present well the dsRNA degrading activities of different insect species and tissues.

Considering that insects usually differ in terms of their physiological states, and that enzyme activity always varies with catalyzing reaction conditions, we hypothesized that not only regulation of enzyme expression, but also the reaction environments of the gut and hemolymph influence dsRNA degradation rates among different insects. Thus, characterizing the physiological conditions of the gut and hemolymph in different insects and pursuing the mechanisms whereby dsRNA-degrading activity is regulated could help us establish a more comprehensive understanding of the major factors that affect dsRNA persistence. This will be helpful in establishing efficient strategies to improve dsRNA stability.

**FIGURE 1:**
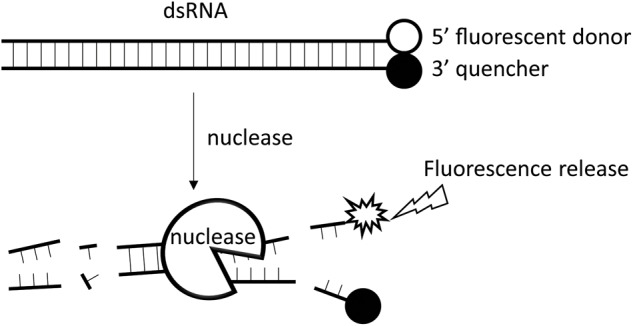
The working principle of the fluorescence method. A 24-bp synthesized dsRNA with a fluorophore group attached to the 3′ end of its sense strand and a quencher group to the 5′ end of its antisense strand is used as the substrate for analysis of dsRNA nucleases. No fluorescence is emitted when the substrate remain intact as the quencher group inhibits the fluorophore group. When dsRNA nuclease is added to the reaction tube, the dsRNA substrate will be gradually degraded, leading to separation of the fluorophore group from the quencher group, and thus the emission of the corresponding fluorescence light. The more the dsRNA degraded, the stronger the fluorescence signal.

Traditionally, dsRNA degrading activities have been assayed by analyzing substrate residues using gel electrophoresis and spectrometer absorbance at OD_260_ (Arimatsu et al., 2007a; Liu et al., 2012). These methods do not allow for precise, quantitative tests. Recently, a more accurate method was developed which analyzes residual dsRNA molecules using quantitative PCR (Garbutt et al., 2013; Wang et al., 2016). This method has the disadvantage of being impractical when large quantities of samples must be analyzed. However, the continuous fluorescence intensity measurement method developed for Dicer cleavage assays is applicable for such analyses (Podolska et al., 2014). Here, we modified the method by selecting a suitable fluorophore and quencher pair and developed a fluorescence method for quantification of dsRNA degrading activity (**Figure 1**).

## Materials and Methods

### Insects

Four species of insects representing different orders were compared in this study. For comparative analysis, the insects used in the following experiments were at the same developmental stage as those used in the previous RNAi experiments: middle 6^th^ instar tobacco caterpillar larvae (*Spodoptera litura*, Lepidoptera), 2-day-old adult migratory locusts (*Locusta migratoria*, Orthoptera), 3-day-old adult American cockroaches (*Periplaneta americana*, Blattaria), and middle 5^th^ instar tenebrionid beetle larvae (*Zophobas atratus*, Coleoptera). These insects were selected because they were all in their gluttonous feeding phase and had similar body weights. Insect rearing conditions are described in our previous study, and their sensitivities to RNAi with both injection and ingestion of dsRNA have been reported (Wang et al., 2016).

### Preparation of dsRNA Substrate

Two substrate types were prepared for dsRNA degrading activity analysis: naked and fluorophore-labeled. For the gel electrophoresis assay, 414 bp of naked dsRNA without fluorophore modification was synthesized using enhanced green fluorescence protein gene (*EGFP*, GenBank accession: DQ389577.1) as template, and using *in vitro* transcription and the T7 RiboMAX Express RNAi System (Promega, Madison, WI, United States). A pair of primers (Forward: 5′-AAGTTCAGCGTGTCCGGC-3′, Reverse: 5′-CACCTTGATGCCGTTCTTC-3′) containing 5′ T7 promoter sites (5′-TAATACGACTCACTATAGGG-3′) targeting *EGFP* was used to generate the DNA template. Purified dsRNA was dissolved in nuclease-free water and stored at -80°C for further use. The quality of all the dsRNA products was checked by gel electrophoresis on 1.2% agarose, and nucleotide concentrations were measured using a NanoDrop ND-1000 spectrophotometer (Thermo Fisher Scientific, Waltham, MA, United States) and adjusted before use. For the fluorescence assay, the fluorescent conjugated dsRNA substrate, which was a 24 bp dsRNA targeting *EGFP* (Sense strand: 5′-ACUUAGCUUAGCACAAACAACCCG-3′, Antisense strand: 5′-CGGGUUGUUUGUGCUAAGCUAAGU-3′), was synthesized by GenePharma Company (Shanghai, China). The 5′ end of the sense strand was labeled with fluorophore and 3′ end of the antisense strand was labeled with quencher.

### Insect Dissection

To collect the hemolymph, gut fluid, gut, and carcass (without gut and hemolymph), the insects were anesthetized on ice prior to dissection. The hemolymph and gut fluid were collected following the procedure described in our previous study (Wang et al., 2016). The guts were dissected and transferred to 1.5 mL micro centrifuge tubes, and then cut into pieces. The gut fluid was obtained by centrifugation at 16,000 × *g* for 10 min at 4°C to remove tissues. Hemolymph was collected with pipettes and transferred to 1.5 mL micro centrifuge tubes. The serum was prepared from the hemolymph by centrifugation at 16,000 × *g* for 10 min at 4°C to remove cells. The remainder of the insect body was collected as the carcass after removing the hemolymph and the gut. After dissection, collected tissues were flash frozen in liquid nitrogen immediately prior to homogenization in 0.1 M Glycine-KOH buffer. Homogenization was performed using 1 g extract in 8 mL 0.1 M Glycine-KOH buffer with specific pH values adjusted according to the requirements of individual experiments. All of the whole-body sample sets contained at least three insect individuals, and those representing guts and hemolymph came from at least five insect individuals. All experiments were performed with three biological replications and all tests with three technical replications.

### Measurement of dsRNA Degrading Activity by Fluorescence Method

Optimal reaction conditions for the enzymes from different insects were determined before further experimentation was carried out. Glycine-KOH buffer (with pH ranging from 6.5 to 11.0) containing 0.1 M glycine, 0.1 M NaCl, 1 mM MgCl_2_, 1 mM phenylthiourea (PTU), 1 mM dithiothreitol (DTT), 1 mM phenylmethanesulfonyl fluoride (PMSF) and 10% glycerol was used as homogenization and reaction buffer when determining optimal pH. Whole insect bodies were homogenized on ice using a glass homogenizer, and the crude enzyme lysate was cleared by centrifugation at 16,000 × *g* for 10 min at 4°C. The supernatant was transferred to a new microcentrifuge tube and centrifuged again at 16,000 × *g* for 20 min at 4°C. The final supernatant was then collected as crude enzyme solution. The dsRNA degradation assays were conducted in black, flat-bottom, polystyrene 384-well microplates (Corning, NY, United States). Reaction mixtures containing 19 μL crude enzyme solution and 1 μL (10 μM) fluorescent conjugated dsRNA substrate were incubated at 37°C. Fluorescence intensity was continuously measured with a Softmax M5 Pro Multi wavelength fluorescence reader (Molecular Devices, Sunnyvale, CA, United States), with readings taken at 30 s intervals for 60 min. To determine the optimal magnesium concentration, 19 μL of crude enzyme solutions containing different concentrations of magnesium (0–64 mM) at the predetermined optimal pH were incubated with 1 μL (10 μM) fluorescent conjugated dsRNA substrate. To determine the optimal temperature, 19 μL crude enzyme solutions containing the optimal magnesium concentration at the optimal pH were incubated with 1 μL (10 μM) fluorescent conjugated dsRNA substrate at a range of temperatures. The rate of dsRNA degradation indicated by changes in Rate of Fluorescence Units (RFU ⋅ s^-1^ or RFU ⋅ mg^-1^ protein ⋅ s^-1^) was calculated based on the initial linear velocity by plotting fluorescence intensity (RFU) against time.

Kinetic parameters of the enzymes from different insects were determined using their individualized optimal reaction conditions. Different concentrations of the fluorescent conjugated dsRNA substrate were incubated with the crude enzyme, prepared as described above, in a total volume of 20 μL. Steady-state kinetic parameters were estimated by measuring the rate of reaction over the linear range with respect to time, with substrate concentrations ranging from 0 to 16 μM. Calculations were performed by fitting the data to the Michaelis–Menten equation using GraphPad Prism 6.03 (GraphPad Software Inc., La Jolla, CA, United States).

### Visualization of dsRNA Degrading Activity by Gel Electrophoresis

Enzyme solutions were prepared by dilution of the gut fluid and serum collected from the tobacco caterpillar with nuclease free water. Both gut fluid and serum were diluted 20-fold. Then, 19 μL of the dilutions were incubated with 1 μL (1 μg) of the 414 bp naked dsRNA at 37°C, and samples were collected at different time points (2, 10, 30, 60, and 120 min, respectively). After collection, 1 μL of proteinase K (Qiagen, Hilden, Germany) was immediately added to the samples, which were incubated at 55°C for 15 min to stop the RNA-degrading reaction and eliminate proteins. The integrity of residual dsRNA was then evaluated by electrophoresis on a 1.5% agarose gel. The bands were visualized with ethidium bromide (EtBr) under ultraviolet light.

### Measurement of dsRNA Degrading Activity by Quantitative Real-Time PCR (qPCR) Analysis

Naked 414 bp dsRNA was used as substrate and incubated with enzyme solutions at 37°C for 5 min in a total volume of 20 μL. Then, 350 μL buffer-RLT from the RNeasy Micro Kit (Qiagen, Hilden, Germany) was added to stop the reaction, and the residual dsRNA was extracted by using this kit. Quantitative measurement of residual dsRNA content was conducted as described previously (Garbutt et al., 2013; Wang et al., 2016). The dsRNA samples were denatured at 65°C for 5 min prior to cDNA preparation by reverse transcription using a PrimeScript^TM^ 1st Strand cDNA Synthesis Kit (TaKaRa, Dalian, China). The residual dsRNA content was quantified using qPCR performed with SYBR^®^ Premix Ex Taq^TM^ reagent (TaKaRa) and Applied Biosystems 7500 System (Life Technologies, Carlsbad, CA, United States). The qPCR reaction mix containing 10 μL SYBR mix, 0.4 μL each of forward and reverse gene-specific primer (Forward: 5′-GACGACGGCAACTACAAGAC-3′, Reverse: 5′-GTCCTCCTTGAAGTCGATGC-3′), 0.4 μL ROX dye reagent of the kit, and 1 μL of cDNA template. Nuclease-free water (7.8 μL) was added to a final volume of 20 μL. The qPCR program was as follows: 95°C for 30 s, 40 cycles at 95°C for 5 s, and 60°C for 34 s. Residual dsRNA content was quantified using the formula derived from the calibration experiments with serially diluted dsRNA in the inactivated gut fluid solution (*y* = -3.340*x* + 34.58, *r*^2^ = 0.9901). Rate of dsRNA degradation was calculated using the following formula: (1,000,000-residual dsRNA)/300, with units expressed as pg⋅s^-1^.

### Measurement of Tissue pH and Magnesium Ion Concentrations

Fresh serum and gut fluid were prepared from the different insect species. The pH values of serum and gut fluids were measured using an InLab^®^ Ultra-Micro pH electrode (Mettler-Toledo, Greifensee, Switzerland) (Harrison, 2001). For magnesium concentration measurement, the serum and gut fluids were first digested with HNO_3_ for 40 min with a microwave digestion system (Milestone Ethos T, Sorisole, BG, Italy), after which digested solutions were transferred to volumetric flasks and diluted with 1% HNO_3_ to a final volume of 50 mL. The magnesium ion concentration in the solutions were measured using inductively coupled plasma-mass spectrometry (ICP-MS) with axial and radial viewing plasma configuration Model Optima 8000 (PerkinElmer, Waltham, MA, United States) (Zhou et al., 2007).

### Protein Quantification

Protein concentrations in crude enzyme solutions were determined by Bradford assay using bovine serum albumin (BSA) as standard. The reaction mixtures, containing 10 μL protein solutions and 270 μL Coomassie brilliant blue, were incubated at 25°C for 5 min and the absorbance subsequently measured at 595 nm with a Softmax M5 Pro Multi wavelength fluorescence reader (Molecular Devices, Sunnyvale, CA, United States).

### Statistical Analysis

Correlation analyses of differences between dsRNA-degrading activity determined by fluorescence and qPCR methods were performed in GraphPad Prism 6.03 (GraphPad Software Inc., La Jolla, CA, United States) using Pearson’s correlation coefficient. The statistical significance of differences in dsRNA degrading activity and in physiological conditions of different insect species were determined by one-way analysis of variance (ANOVA) followed by Tukey’s test using SPSS Statistics 20.0 software (IBM, NY, United States).

## Results

### Development of a Method for Enzymatic Analysis of dsRNA Degrading Nucleases

The continuous fluorescence intensity measurement method developed for the Dicer cleavage assay was first reported by Podolska et al. (2014). The principle is illustrated in **Figure 1**.

#### Fluorophore/Quencher Selection

**FIGURE 2:**
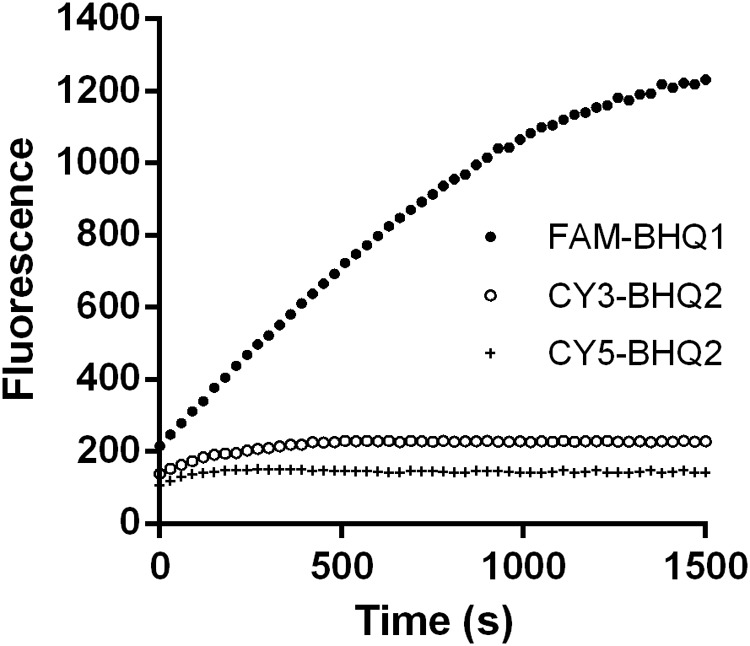
Degradation time course of a 24-bp EGFP dsRNA substrate labeled with different fluorophores and quenchers. Three different combinations of fluorophore and quencher types were considered: 5-FAM fluorescent donor and 3-BHQ1 quencher-labeled strands (5-FAM/3-BHQ1), 5-Cy3 fluorescent donor and 3-BHQ2 quencher-labeled strands (5-Cy3/3-BHQ2), and 5-Cy5 fluorescent donor and 3-BHQ2 quencher-labeled strands (5-Cy5/3-BHQ2). The gut fluid from *S. litura* was 20-fold diluted (1 μL fluid + 19 μL nuclease-free water) and incubated with 1 μL of a 24-bp EGFP dsRNA labeled with different fluorophore/quencher combination (final concentration 0.5 μM) at 37°C. The florescence signal resulted from degradation of the dsRNA was monitored at 30 s intervals from 0 to 1500 s.

Suitable fluorophore and quencher couples were selected for quantification of dsRNA degradation rate. Three different combinations of fluorophore and quencher types were considered: 5-FAM fluorescent donor and 3-BHQ1 quencher-labeled strands (5-FAM/3-BHQ1), 5-Cy3 fluorescent donor and 3-BHQ2 quencher-labeled strands (5-Cy3/3-BHQ2), and 5-Cy5 fluorescent donor and 3-BHQ2 quencher-labeled strands (5-Cy5/3-BHQ2). When tobacco caterpillar gut fluid was used as the nuclease source and incubated with the 24 bp dsEGFP substrates labeled with these fluorescent and quencher combinations at a final concentration of 0.5 μM dsRNA, the fluorescence intensity was measured at excitation and emission wavelengths of 494 and 519 nm for fluorophore FAM, 552 and 570 nm for fluorophore Cy3, and 650 and 670 nm for fluorophore Cy5, respectively. As shown in **Figure 2**, the fluorescence intensity in the reaction with 5-FAM/3-BHQ1-labeled substrate continuously increased from 215.33 to a maximum of 1232.15 in 25 min. In the reaction with 5-Cy3/3-BHQ2 and 5-Cy5/3-BHQ2-labeled substrates, the fluorescence intensity increase was less than 2-fold and reached the maximum in 5 min. The 5-FAM/3-BHQ1 combination showed long-lasting continuous changes in fluorescence and a high fluorescence yield, and was thus the best candidate for further experiments.

#### Fluorescent Method vs. Gel Electrophoresis

**FIGURE 3:**
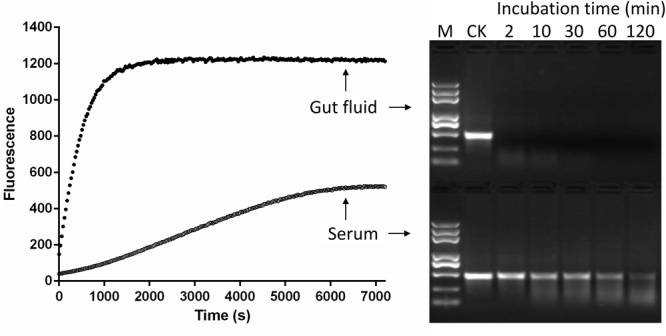
Comparison of fluorescent **(Left)** and gel electrophoresis **(Right)** methods for analyzing dsRNA degrading nucleases in *S. litura* serum and gut fluid. Both serum and gut fluid were diluted 20 times with nuclease free water, and the fluorescence reaction was incubated with 24 bp fluorescence labeled dsEGFP in the final concentration 0.5 μM. The gel electrophoresis reaction was incubated with 414 bp naked dsEGFP in the final concentration 0.05 μg/μL. CK: Control without enzymes. M: Trans 2K Plus DNA marker. Numbers 2–120 were the minutes indicating sample incubation time.

The traditional agarose gel electrophoresis assay was used to check whether the fluorescence method using the 5-FAM/3-BHQ1-labeled 24 bp dsEGFP substrate was suitable for the detection of dsRNA degradation. The serum and gut fluids from *S. litura* were incubated with 5-FAM/3-BHQ1-labeled 24 bp dsEGFP, and the reaction was monitored using the fluorescence monitoring method. A parallel control degradation experiment was performed using 414 bp dsEGFP as substrate and the reaction was monitored by checking the residual substrate with gel electrophoresis. As shown in **Figure 3**, both detection methods yielded similar results. In the reaction with gut fluid, the saturation of fluorescence intensity took about 2100 s (35 min) and the band of residual dsRNA on the gel disappeared within 30 min. With serum, the fluorescence intensity increased only slightly (less than half the saturation in the gut fluid reaction) over the course of 2 h, and in the parallel experiment for gel electrophoresis method, the band of residual dsEGFP on the gel was still clearly visible after 2 h of incubation. This result indicated that the newly developed fluorescence method delivered accurate results and was obviously superior to gel electrophoresis, was easier to perform, and could be used for straightforward and reliable quantification of dsRNA.

#### Fluorescent Method vs. qPCR

**FIGURE 4:**
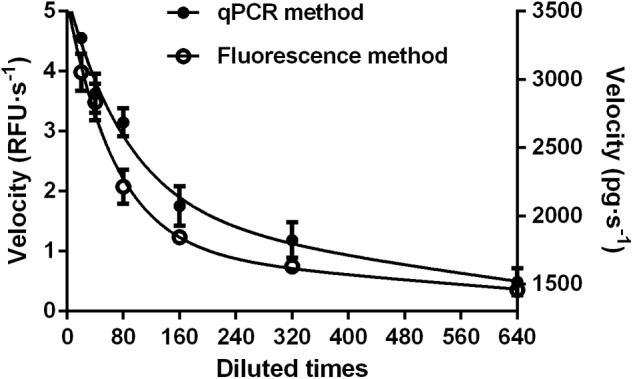
Comparison of fluorescent **(Left)** and qPCR **(Right)** methods for analyzing dsRNA degrading nucleases in *S. litura* gut fluid. Serial dilutions of the gut fluid (20, 40, 80, 160, 320, and 640 times) were incubated with 1 μL 24 bp fluorescence labeled dsEGFP or 1 μL 414 bp naked dsEGFP in a total volume of 20 μL at 37°C. The final substrate concentration was 0.5 μM for fluorescence method and 0.05 μg/μL for qPCR method. Values are mean ± SE; *n* = 3.

The fluorescence method was also validated by monitoring dsRNA degrading activity of serially diluted gut fluid from *S. litura*, using a well-accepted qPCR method. The parallel experiments were set up using identical enzyme solutions, but different substrates. As shown in **Figure 4**, the two methods produced similar curves with a correlation coefficient of 0.976 (*P* = 0.0009). This result further validated the fluorescence method’s effectiveness as being similar to that of the qPCR method for detecting dsRNA degrading activity. Moreover, it has the benefit of being a comparatively easier method.

### Biochemical Differences of the dsRNA Degrading Nucleases in Four Insects

#### Impact of pH

**FIGURE 5:**
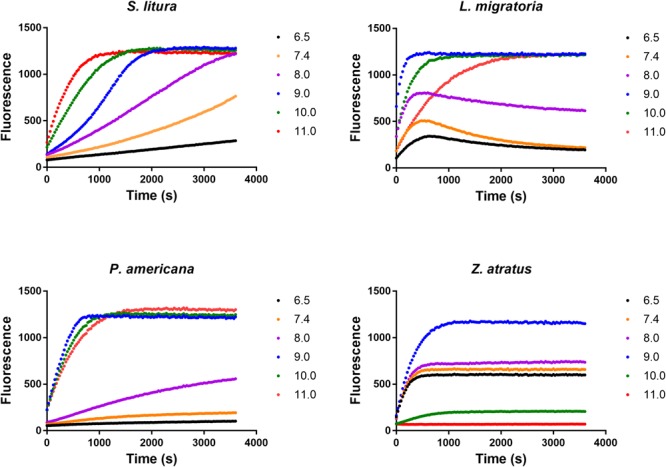
Distinct impacts of pH on the dsRNA degrading nucleases of four insects. The homogenates were prepared from whole body of different insects. The reaction buffer containing 0.1 M Glycine, 0.1 M NaCl, 1 mM MgCl_2_, 1 mM PTU, 1 mM DTT, 1 mM PMSF and 10% Glycerol was adjusted by KOH to different pH (6.5, 7.4, 8.0, 9.0, 10.0, and 11.0). Each reaction containing 19 μL enzyme solution and 1 μL fluorescence labeled dsRNA substrate at a final concentration of 0.5 μM. The fluorescence intensity in different reactions were continuously monitored at 37°C. Values are mean of fluorescence intensity; *n* = 3.

The effect of pH on dsRNA degrading activity was measured in Glycine-KOH buffer (pH 6.5, 7.4, 8.0, 9.0, 10.0, and 11.0). Enzymes from four insect species were tested, and the optimal pH for three of them was found to be 9.0 (**Figure 5**). However, the enzyme from *S. litura* was singular in that it displayed its highest activity at a pH of 11.0, the upper limit of the test range. This confirmed the assertion that degradation of dsRNA in different insects is alkaline-activated. Besides this, the intimate neighbor relationship between the two curves of pH 6.5 and pH 7.4 implied that small variations within the neutral pH range did not overtly influence enzyme activity, and that the influence of pH was mainly found under alkaline conditions (pH 7.4–11.0), highlighting the differences between insects. Other than *S. litura, P. americana* also retained high activity at pH 10.0 and pH 11.0. However, when pH exceeded the optimal range (pH 9.0), activities decreased markedly in the other two insect species, and especially for *Z. atratus*. In the following studies, considering pH 11.0 is extremely alkaline and that dsRNA is structurally unstable under such conditions (Christiaens and Smagghe, 2014), pH 10.0 was used instead of the optimal pH for the *S. litura* enzyme.

#### Impact of Mg^2+^ Concentration

**FIGURE 6:**
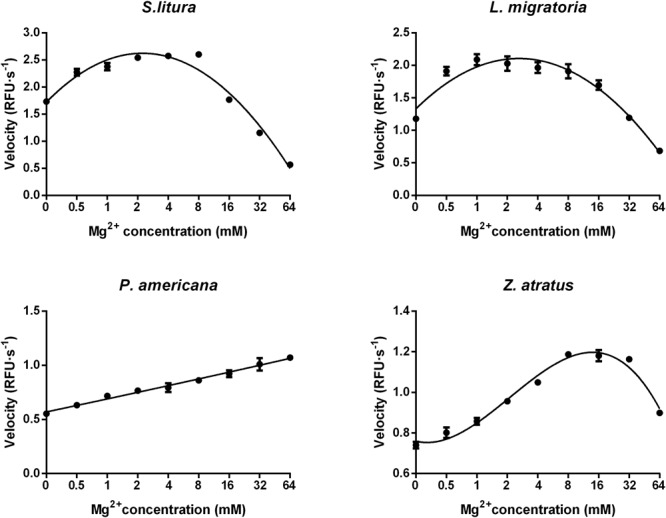
Distinct impacts of Mg^2+^ concentrations on the dsRNA degrading nucleases of four insects. The homogenates were prepared from whole body of different insects. Tested conditions were conducted under their optimal pH (pH 10.0 for *S. litura* and pH 9.0 for the other three species). Each reaction containing 19 μL enzyme solution and 1 μL fluorescence labeled dsRNA substrate at a final concentration of 0.5 μM. The fluorescence intensity in different reactions were continuously monitored at 37°C. Values are mean ± SE; *n* = 3.

Optimization of Mg^2+^ concentration was conducted at the optimal pH (pH 10.0 for *S. litura*, pH 9.0 for *L. migratoria, P. americana*, and *Z. atratus*) using the reaction buffer containing different concentrations of MgCl_2_. As shown in **Figure 6**, an optimal amount of Mg^2+^ could stimulate dsRNA degrading activity in all four of the insect species tested. The optimal Mg^2+^ concentration range for dsRNA degrading activity in *S. litura* and *L. migratoria* was 0.5–8 mM, and for *Z. atratus* it was 8–32 mM. However, no optimal Mg^2+^ concentration range was found for *P. americana*, whose dsRNA degrading ability slowly increased (by 2-fold in total) with increasing Mg^2+^ concentration within the range tested (0–64 mM). Considering the limited changes in activity over a large range of Mg^2+^ concentrations among different insect enzymes, 8 mM is likely to be an optimal Mg^2+^ concentration for most insects.

#### Impact of Temperature

**FIGURE 7:**
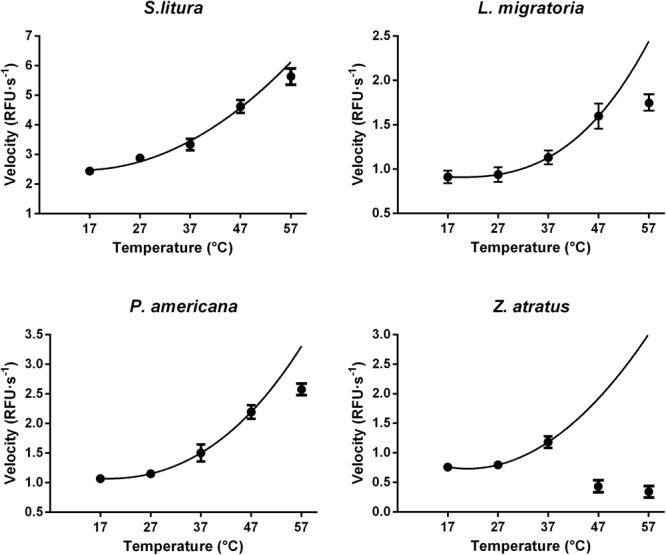
Distinct impacts of temperature on the dsRNA degrading nucleases of four insects. The homogenates were prepared from whole body of different insects. The test reaction used the buffer with optimal pH and Mg^2+^ concentrations for different insect species (pH 10.0 and 8 mM Mg^2+^ for *S. litura*, pH 9.0 and 8 mM Mg^2+^ for the other three species). Each reaction containing 19 μL enzyme solution and 1 μL fluorescence labeled dsRNA substrate at a final concentration of 0.5 μM. The fluorescence intensity in different reactions were continuously monitored at different temperatures. Values are mean ± SE; *n* = 3.

Suitable reaction temperatures were determined under optimal pH and Mg^2+^ concentration conditions (pH 10.0 and 8 mM Mg^2+^ for *S. litura*, pH 9.0 and 8 mM Mg^2+^ for *L. migratoria, P. americana*, and *Z. atratus*). As expected, the initial dsRNA degrading activity increased with temperature at lower temperatures following the well-known temperature effect rule (*Q*_10_), but was inhibited at higher temperatures. In *S. litura, L. migratoria*, and *P. americana* the activity increased smoothly between 17 and 47°C, after which the rate of increase slowed down (**Figure 7**). In *Z. atratus*, the enzyme was extremely sensitive to high temperatures, with the activity dropping sharply above 37°C. Thus, 37°C may be a suitable temperature for higher enzyme activity in most insects. Besides, as shown in **Figure 7**, the slope of the temperature/activity curve varied in different insect species (*Q*_10_ range: 1.21–1.28). This also means that dsRNA degrading enzymes in different insects may vary marginally in their sensitivity to temperature.

#### Saturation Curves and Enzyme Kinetics

**FIGURE 8:**
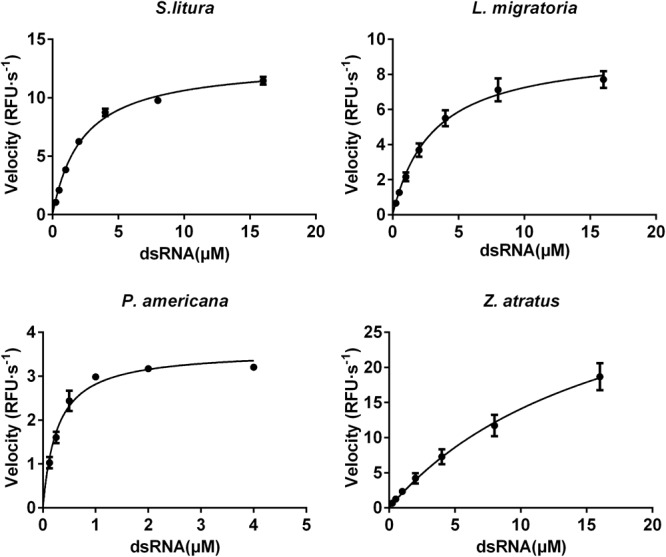
Comparison of the saturation curves of the dsRNA degrading nuclease of four insects. The homogenates were prepared from whole body of different insects. The test reaction used the buffer with optimal pH and Mg^2+^ concentrations for different insect species (pH 10.0 and 8 mM Mg^2+^ for *S. litura*, pH 9.0 and 8 mM Mg^2+^ for the other three species). Different concentration of dsRNA substrates were incubated with 19 μL enzyme solutions in a total volume of 20 μL. The fluorescence intensity in different reactions were continuously monitored at 37°C. Values are mean ± SE; *n* = 3.

The *K*_m_ and *V*_max_ of dsRNA degrading enzymes from different insect species were calculated using non-linear regression to fit the plot of velocities against substrate concentrations (**Figure 8**). The enzyme from *P. americana* exhibited high substrate affinity (*K*_m_ 0.27 μM) and low capacity (*V*_max_ 3.58 μM⋅s^-1^). Whereas *Z. atratus* showed low affinity (*K*_m_ 17.59 μM) but high capacity (*V*_max_ 38.87 μM⋅s^-1^). The *K*_m_ values of *S. litura* and *L. migratoria* enzymes were 2.28 and 3.06 μM, respectively, and the corresponding *V*_max_ values were 13.40 μM⋅s^-1^ and 9.46 μM⋅s^-1^, respectively. For the enzymes from the four insects tested, the *K*_m_ values ranked as follows: *P. americana* 0.27 < *S. litura* 2.28 ≈*L. migratoria* 3.06 < *Z. atratus* 17.59 and the *V*_max_ *P. americana* 3.58 < *S. litura* 13.4 ≈*L. migratoria* 9.46 < *Z. atratus* 38.87, but were of similar order. It appears that different insects maintain proper dsRNA degrading activity by balancing the *K*_m_ and *V*_max_ of their enzymes.

### Differences in the Tissue Distribution of dsRNA Degrading Nucleases Among Four Different Insect Species

The dsRNA degrading activity in insect whole body, gut, hemolymph and other tissues (carcass) was evaluated in each species at their own experimentally determined optimal pH and Mg^2+^ concentrations. As shown in **Table 1**, the highest activity was found in gut tissue, and was over 100-fold higher than the activity in hemolymph and carcass in all four of the insect species tested (**Table 1**). When different insects were compared, whole body activity varied significantly between species. In terms of tissue activity, *L. migratoria* had extremely high activity in its gut. However, its hemolymph activity was relatively low, being only slightly higher than that of *P. americana* and *Z. atratus. S. litura* not only had higher activity in its gut, but also had much higher activity in its hemolymph than the other insects tested. *P. americana* had low activity in its gut and very low activity in its hemolymph. *Z. atratus* had the lowest overall activity in its tissues of all the tested species. Whole body activity of different insects could be ranked in the following order: *L. migratoria* > > *P. americana* ≥*S. litura > > Z. atratus*, which is completely different from their RNAi sensitivities: *P. americana* > *Z. atratus* > > *L. migratoria* > > *S. litura.* This inconsistence may result from other factors influencing RNAi efficiency, and also imply that the dsRNA degrading enzymes function *in vivo* at the conditions different from those we used for activity tests.

**Table 1:** Spatial distribution patterns of dsRNA degrading nucleases in four insect species^†^ (RFU⋅mg^-1^ protein⋅s^-1^).

Insects	Whole body	Gut	Hemolymph	Carcass
*Spodoptera litura*	28.2 ± 8.4b	1767.1 ± 371.5b	8.8 ± 0.3a	0.9 ± 0.2b
*Locusta migratoria*	3019.4 ± 25.5a	119081.8 ± 10596.1a	0.4 ± 0.1b	218.7 ± 6.8a
*Periplaneta americana*	48.1 ± 16.9b	756.4 ± 122.1c	0.13 ± 0.01c	1.2 ± 0.3b
*Zophobas atratus*	8.5 ± 1.7c	34.66 ± 3.98d	0.13 ± 0.05c	0.28 ± 0.05c

*^†^Tested under optimal pH and Mg^*2*+^ concentrations for each insect species. pH 10.0 and 8 mM Mg^*2*+^ for S. litura, pH 9.0 and 8 mM Mg^*2*+^ for the other three.*

*Different letters (a, b, c, d) indicate significant differences among insects (P < 0.05). Values are mean ± SD; n = 3.*

### Differences in Physiological Conditions Among Four Insect Species

The pH and Mg^2+^ concentration in serum and gut fluids from four insect species were tested. The results presented in **Table 2** indicate that these insects differed in terms of their internal chemical environments. Serum pH appeared neutral with minor variation, ranging from 6.69 to 7.16, depending on the insect species. The gut fluids of *L. migratoria, P. americana*, and *Z. atratus* were slightly acidic, with pH ranging from 5.74 to 6.23. However, the gut fluid of *S. litura* was alkaline with a pH of 8.72. Mg^2+^ concentrations in both the serum and gut fluid also varied among the four insect species. *S. litura* had high serum Mg^2+^ (39.37 mM), but extremely low gut fluid concentrations (2.75 mM). In comparison, *L. migratoria* Mg^2+^ concentration was low in serum (10.68 mM), but high in gut fluid (40.45 mM). *P. americana* was found to have low Mg^2+^ concentrations in both its serum (4.64 mM) and gut fluid (11.39 mM). For *Z. atratus*, high Mg^2+^ concentration was found in both serum (35.36 mM) and gut fluid (42.54 mM). Neither the serum nor the gut fluids of any of the insects tested were optimal for *in vitro* dsRNA degradation when both pH and Mg^2+^ concentrations were taken into consideration.

**Table 2:** Measurements of pH values and Mg^2+^ concentrations in the gut lumen and hemolymph of four insect species.

Insects	pH		Mg^2+^(mM)	
	Serum	Gut fluid	Serum	Gut fluid
*Spodoptera litura*	6.69 ± 0.02b	8.72 ± 0.12a	39.37 ± 0.94a	2.75 ± 0.19c
*Locusta migratoria*	6.82 ± 0.20b	5.79 ± 0.25c	10.68 ± 0.47c	40.45 ± 1.37a
*Periplaneta americana*	7.16 ± 0.13a	6.23 ± 0.08b	4.64 ± 0.23d	11.39 ± 0.64b
*Zophobas atratus*	6.84 ± 0.09b	5.74 ± 0.04c	35.36 ± 1.37b	42.54 ± 2.53a

*Different letters (a, b, c, d) indicate significant differences among insects (P < 0.05). Values are mean ± SD; n = 3.*

### Comparison of the Optimal and Physiological Activity of dsRNA Degrading Nucleases Among Four Different Insects

The serums and gut fluids of different insects were compared in terms of their dsRNA degrading activity under physiological and optimal pH and Mg^2+^ concentration conditions. The results in **Table 3** clearly show that both gut fluids and serums from different insect species exhibited much lower dsRNA degrading activities under physiological conditions than under their respective optimal conditions in most cases (4.5–378.2-fold differences). However, the gut fluid of *S. litura* (1.6-fold) and the serum of *Z. atratus* (1.0-fold) did not notably deplete degradative activity. Correspondingly, the pH in the gut fluid of *S. litura* was higher than in other species, and the Mg^2+^ concentration in the serum of *Z. atratus* was extremely high.

**Table 3:** Comparison of the optimal and physiological dsRNA nuclease activity in the gut lumen and hemolymph of four insect species (RFU⋅mg^-1^ protein⋅s^-1^).

Insects	Gut fluid			Serum		
	Optimal^†^	Physiological^‡^	Ratio^§^	Optimal	Physiological	Ratio
*Spodoptera litura*	11364.1 ± 777.3b ±	6923.6 ± 109.8a	1.6 ±	30.40 ± 8.72a	0.80 ± 0.20a	38.0
*Locusta migratoria*	18666.3 ± 3580.9a±	49.36 ± 2.62b	378.2 ±	4.47 ± 0.79b	0.66 ± 0.30a	6.8
*Periplaneta americana*	1674.1 ± 68.8c ±	54.72 ± 3.68b	30.6 ±	1.62 ± 1.16c	0.12 ± 0.04b	13.5
*Zophobas atratus*	71.50 ± 3.99d ±	15.72 ± 1.29c	4.5 ±	0.04 ± 0.02d	0.04 ± 0.01c	1.0

*^†^Tested under the optimal pH and Mg^*2+*^ concentrations for each insect species. pH 10.0 and 8 mM Mg^*2+*^ for S. litura, and pH 9.0 and 8 mM Mg^*2+*^ for the other three.*

*^‡^Tested under physiological pH and Mg^*2+*^ concentrations for each insect species. The combination of pH and Mg^*2+*^ concentrations were used as indicated in **Table 2** for gut fluid or serum.*

*^§^Fold changes of enzyme activity under optimal condition against physiological condition.*

*Different letters (a, b, c, d) indicate significant differences among insects (P < 0.05). Values are mean ± SD; n = 3.*

## Discussion

In this study, the continuous fluorescence intensity measurement method developed by Podolska et al. (2014) for Dicer cleavage assays was adopted and modified for monitoring dsRNA degrading activity. Owing to different combinations of fluorophores and quenchers with varying fluorescent intensity (Podolska et al., 2014), selection experiments were performed, which found 5-FAM fluorescent donor- and 3-BHQ1 quencher-labeled 24 bp dsRNA to be the most sensitive substrate. With this substrate, the fluorescence method was validated using traditional gel electrophoresis and a well-accepted micro-quantitative PCR method. The monitoring of dsRNA degrading activity in different insect tissues and series dilutions of gut fluid all showed that the fluorescence method gave reliable results. Furthermore, the fluorescence method is easier to execute than the qPCR method, and more accurate than gel electrophoresis.

With the developed fluorescence method, dsRNA degrading activities in different insects were tested and their characteristics compared. The results clearly showed that insect dsRNA degrading nucleases are basophilic with an optimal pH of 9.0 or more. Furthermore, optimal Mg^2+^ ion concentrations enhanced the activity of nucleases in all of the insect species tested. These results were consistent with previous reports in which purified dsRNase from the digestive juice of *Bombyx mori* (BmdsRNase), and expressed recombinant LmdsRNase2 from *L. migratoria*, were both found to have higher activities under alkaline conditions (Arimatsu et al., 2007a; Song et al., 2017), which were further promoted by addition of divalent cations (Arimatsu et al., 2007a). Furthermore, our results showed that the enzymes’ optimal pH, Mg^2+^ concentration range, and temperature varied with insect species. Enzymes from *S. litura*, a defoliator feeding on alkaline food, had the highest optimal pH. BmdsRNase from another lepidopteran defoliator has been reported to also have pH-dependent activity (Arimatsu et al., 2007a). The enzyme from *Z. atratus*, a store grain pest living in shedding rooms, was highly sensitive to high temperatures, and its activity was greatly inhibited above 37°C. For the other insects living in open air, enzymes were inhibited at temperatures higher than 47°C. It appears that different insects produce diverse dsRNA degrading enzymes with different properties. Whether these properties are coupled with their habitats requires further investigation.

The different kinetic parameters of enzymes, tested under the optimal conditions of each enzyme, not only provided further confirmation that different insects produce different nucleic acid degrading enzymes, but also demonstrated that they pcteristic parameter of enzymes, *K*_m_, was found to differ in enzymes from *P. americana* (0.27 μM), *S. litura* (2.28 μM), *L. migratoria* (3.06 μM), and *Z. atratus* (17.59 μM). Although tested with whole body extracts, and as a weighted average integrating all enzymes, the variation in *K*_m_ could indicate different production profiles for nucleic acid degrading enzymes in each insect. Consistent with this finding, previous identification of gut-specific double-stranded RNA degrading enzymes proved that the dsRNase activity was mostly attributable to genes encoding DNA/RNA non-specific endonuclease family enzymes (Wynant et al., 2014). The knockdown effect of dsRNA degrading nuclease activity in different insects also demonstrated the existence of a variety of enzymes in different insects (Luo et al., 2017; Song et al., 2017; Spit et al., 2017). *V*_max_ is a parameter depending on the enzyme protein concentration. Thus, different *V*_max_ values displayed by the enzymes of different insects (*P. americana* 3.58 μM⋅s^-1^, *S. litura* 13.4 μM⋅s^-1^, *L. migratoria* 9.46 μM⋅s^-1^ and *Z. atratus* 38.87 μM⋅s^-1^) might indicate that these insects had different enzyme concentrations in their bodies. An interesting observation was that, among different insects, higher enzyme affinity (lower *K*_m_) was usually accompanied with lower enzyme quantity (lower *V*_max_). It seems this is an evolutionary adaptation for maintaining enzyme activity at the required level.

Whole-body dsRNA degrading activities measured under optimal conditions (pH and Mg^2+^ concentrations) did not correspond well to previously reported RNAi sensitivities (Wang et al., 2016). Previous work showed that *P. americana* and *Z. atratus* were both equally sensitive to injection and ingestion RNAi; *L. migratoria* was sensitive to injection RNAi, but not to ingestion; and *S. litura* was insensitive to both injection and ingestion RNAi (Wang et al., 2016). The order of gut activity, *L. migratoria* > > *S. litura* > *P. americana* > > *Z. atratus*, was a slightly better indicator of their susceptibility to ingestion RNAi. Insects’ hemolymph activity, *S. litura* > > *L. migratoria* > *P. americana* ≈*Z. atratus*, was comparable to their injection RNAi sensitivity. These results indicated that whole-body activity was not a good indicator for RNAi efficiency, but that hemolymph and gut fluid activities are acceptable indicators for injection and ingestion RNAi, respectively. Data showed that the dsRNA-degrading activity in tissues which contact the delivered dsRNA first was much higher than whole-body activity. This means that the gut is important for degradation of ingested dsRNA, and hemolymph, for injected dsRNA. This is consistent with previous reports (Luo et al., 2013; Wang et al., 2016). However, RNAi efficiency is not totally dependent on dsRNA permanence. Though little evidence was obtained, the sensitivity of RNAi core machinery and dsRNA transportation might involve (Tomoyasu et al., 2008; Huvenne and Smagghe, 2010). Otherwise, we analyzed the enzymes with the data tested *in vitro*, finding that they may work optimally under different conditions.

Therefore, we measured the pH and Mg^2+^ concentrations in the gut lumen and hemolymph of the four insects, finding that their pH and Mg^2+^ concentrations were not optimal for their dsRNA degrading enzymes. Our work has demonstrated that insect dsRNA degrading enzymes are basophilic. However, in most insect species, hemolymph pH commonly ranges from neutral to slightly acidic (Wyatt, 1961). Here the tested hemolymph pH in four insect species ranged from 6.69 to 7.16. This means that the pH of hemolymph could place limitations on high enzyme activity, and its narrow near-neutral variable range had a negligible effect on dsRNA degrading activity, as the results indicated. The varied Mg^2+^ concentrations may play a relatively greater role in regulation of enzyme activity in hemolymph. The pH of insect midguts is reported to range widely from acidic to alkaline (Johnson and Felton, 1996). Our results showed that the gut fluids of *L. migratoria, P. americana*, and *Z. atratus* were typically acidic to neutral. In the caterpillar *S. litura*, the pH was extremely alkaline, reaching pH 8.72. Thus, dsRNA degrading activity of *S. litura* extract tested at gut fluid pH was similar to that at optimal pH, while activities in the other three insects were depressed dramatically. These findings imply that physiological reaction conditions can regulate dsRNA degrading activity in various insects, and modify their RNAi sensitivity.

Tissue activities in the four insect species tested under physiological conditions were not better correlated with RNAi efficacy than when tested under optimal conditions. *P. americana* and *L. migratoria* had similar physiologically tested gut fluid activities, and *S. litura* and *L. migratoria* had similar physiologically tested serum activities. This implies that the activities tested in prepared serum and gut fluids are not proportional to the negative dsRNA degrading activities naturally occurring in hemolymph and gut tissues. Previous work has reported that the dsRNA degrading activity of gut fluid may come primarily from excreted dsRNases and should repress RNAi (Almeida Garcia et al., 2017; Luo et al., 2017; Song et al., 2017; Spit et al., 2017). However, gut structure differs between insect species, and especially between those with different feeding habits. It has been established that insect guts vary in terms of pH and chemical environment in areas such as foregut, midgut, hindgut, and their various subdivided chambers. Thus, activities tested in a composite gut fluid mixture cannot accurately represent the true degradation happening in the gut. Thus, the phytophagous locust and the omnivorous cockroach showed different ingestion RNAi sensitivity, despite their dsRNA degrading activity tested in gut fluid under physiological conditions being similar. Serum should be more uniform because of its quick circulation. However, little is known about dsRNA degrading enzymes in serum. Based on good consistency of injection RNAi efficacy, we deduced that the activities in serum might also come mainly from dsRNases. However, we could not rule out the possibility of participation by other, unknown, enzymes. Rapid cellular absorption may be another influencing factor because all insect organs float in the serum. That explains why the serum dsRNase activity was not exactly consistent with injection RNAi tendency among different insect species. As is generally known, pH and Mg^2+^ concentrations vary not only among tissues of different insects, but also in the various subcellular spaces, such as lysosomes and vacuoles, in individual insects. This phenomenon might indicate another regulation mechanism for dsRNA degrading activity, and this regulation seemed so delicate that it was difficult to determine an appropriate activity parameter for describing the exact RNAi sensitivities of different insects.

It should be noted that homogenate supernatants contain a variety of dsRNA degrading enzymes, and that their activities and characteristics should therefore be integrated using a weighted average. The obtained data clearly indicated that all insects tested showed dsRNA degrading activity in their gut which was orders of magnitude higher than in other tested tissues, and this affected the baseline activity for their whole bodies. This indicates that overexpression of dsRNases in the gut lumen is a major negative regulator of the RNAi machinery. This observation has been universally reported in all insect species studied previously (Arimatsu et al., 2007b; Liu et al., 2012; Wynant et al., 2014; Luo et al., 2017; Song et al., 2017; Spit et al., 2017). Thus, our results might reflect the activities and characteristics of dsRNases over-expressed in the gut, although Dicers and other enzymes encoded by unidentified genes may also contribute to the observed effects. The major dsRNases over-expressed in the gut may be key in RNAi efficiency and should be the focus of future studies.

Along with the development of our fluorescence method, we have conducted a comprehensive analysis of the biochemical properties of dsRNA degrading nucleases in four insect species from different orders. Our results revealed the special properties of insect dsRNA degrading enzymes and demonstrated that different insects produce a variety of dsRNA degrading enzymes in different quantities. Furthermore, insects had different physiological conditions in different tissues, which served to modulate enzyme activity. The RNAi tolerance of caterpillar *S. litura* not only resulted from quantitative production of dsRNA degrading enzymes, but also from the alkaline environment of its gut. dsRNA degrading activity may be used to estimate RNAi sensitivity among different insect species, but its use is hindered by the fact that the various methods for testing activity do not precisely mirror the natural physiological conditions found within insects, even ignoring other potentially confounding factors.

## Author Contributions

YP, KW, and ZH designed the research and wrote the paper. YP performed all of the experiments with the help of WF and CS. YP and ZH analyzed the data. All authors read and approved the final manuscript.

## Conflict of Interest Statement

The authors declare that the research was conducted in the absence of any commercial or financial relationships that could be construed as a potential conflict of interest.
